# The non-vesicle cell-free DNA (cfDNA) induces cell transformation associated with horizontal DNA transfer

**DOI:** 10.1007/s11033-023-09016-w

**Published:** 2024-01-22

**Authors:** D. A. De La Cruz-Sigüenza, J. P. Reyes-Grajeda, M. A. Velasco-Velázquez, C. Trejo-Becerril, E. Pérez-Cárdenas, A. Chávez-Blanco, L. Taja-Chayeb, G. Domínguez-Gómez, M. P. Ramos-Godinez, A. González-Fierro, A. Dueñas-González

**Affiliations:** 1 Subdirection of Basic Research, Instituto Nacional de Cancerología (INCan), Tlalpan, 14080 Mexico City, Mexico; 2 Protein Structure Laboratory, Instituto Nacional de Medicina Genomica (INMEGEN), Tlalpan, 14610 Mexico City, Mexico; 3 Department of Pharmacology, Faculty of Medicine, Universidad Nacional Autónoma de México (UNAM), Coyoacan, 04510 Mexico City, Mexico; 4 Department of Pathology, Instituto Nacional de Cancerología (INCan), Tlalpan, 14080 Mexico City, Mexico; 5 Department of Genomic Medicine and Environmental Toxicology, Institute of Biomedical Research, Universidad Nacional Autonoma de Mexico (UNAM), Av. Universidad 3004, Copilco Universidad, Coyoacan, 04510 Mexico City, Mexico

**Keywords:** Cell free nucleic acids, Oncogenic transformation, Extracellular vesicles, Virtosome

## Abstract

**Background:**

Cell-free DNA (cfDNA) is a source for liquid biopsy used for cancer diagnosis, therapy selection, and disease monitoring due to its non-invasive nature and ease of extraction. However, cfDNA also participates in cancer development and progression by horizontal transfer. In humans, cfDNA circulates complexed with extracellular vesicles (EV) and macromolecular complexes such as nucleosomes, lipids, and serum proteins. The present study aimed to demonstrate whether cfDNA not associated with EV induces cell transformation and tumorigenesis.

**Methods:**

Supernatant of the SW480 human colon cancer cell line was processed by ultracentrifugation to obtain a soluble fraction (SF) and a fraction associated with EV (EVF). Primary murine embryonic fibroblast cells (NIH3T3) underwent passive transfection with these fractions, and cell proliferation, cell cycle, apoptosis, cell transformation, and tumorigenic assays were performed. Next, cfDNA was analyzed by electronic microscopy, and horizontal transfer was assessed by human mutant *KRAS* in recipient cells via PCR and recipient cell internalization via fluorescence microscopy.

**Results:**

The results showed that the SF but not the EVF of cfDNA induced proliferative and antiapoptotic effects, cell transformation, and tumorigenesis in nude mice, which were reduced by digestion with DNAse I and proteinase K. These effects were associated with horizontal DNA transfer and cfDNA internalization into recipient cells.

**Conclusions:**

The results suggest pro-tumorigenic effects of cfDNA in the SF that can be offset by enzyme treatment. Further exploration of the horizontal tumor progression phenomenon mediated by cfDNA is needed to determine whether its manipulation may play a role in cancer therapy.

**Supplementary Information:**

The online version contains supplementary material available at 10.1007/s11033-023-09016-w.

## Introduction

Since the existence of cell-free DNA (cfDNA) in healthy individuals was reported by Mandel and Metais in 1948 [1], and Leon and Shapiro in 1977 reported that patients with advanced cancer had higher levels of cfDNA compared with cancer-free individuals [2], the field of cfDNA, also known as circulating DNA, has gained momentum. Currently, the field has expanded to cover several disease conditions where increased levels of cfDNA are commonly observed [3]. In the study of oncology, analyses are aimed at exploiting its diagnostic capabilities such as ‘liquid biopsy’, since most studies, including those using next-generation sequencing, unequivocally have demonstrated that nuclear DNA (cf-nDNA) is well represented in the cfDNA found in plasma/serum [4]. Owing to its non-invasive nature and simple extraction, exDNA in the bloodstream is commonly used for diagnosis, therapy selection, and monitoring. It seems that cfDNA’s role in human physiology and pathology is more multifaceted than previously thought, as it is involved in cell signalling, oxidative stress, coagulation, immunity, and carcinogenesis [5].

The identification and further characterization of extracellular vesicles (EV) have evidenced that cfDNA is also present in these extracellular membrane-containing structures [6, 7]. EVs are a diverse population of biological particles ranging from 30 to 1000 nm [8, 9]. Depending on their origin, biogenesis, and size, EVs are categorized into exosomes, microvesicles, microparticles, and apoptotic bodies [10]. However, the term EV is more commonly used in a broader sense, since isolating one specific subcategory remains technically challenging [11].

In cancer, it has been demonstrate that cfDNA is able to move to other cells in vitro and in vivo, (horizontal DNA transfer), and can confer biological changes to recipient cells [12]. This is not unexpected, since the cfDNA shed from malignant cells contains genetic information that can be transcribed and traduced in recipient cells in vitro and in vivo [13]. Although these convincing evidence, there is little information about the mechanisms by which cfDNA is released from malignant cells and enters recipient cells. Moreover, the structural form of this cfDNA in circulation is yet to be determined. Research from our group and other researchers have shown that cfDNA (supernatant and plasma/serum), through passive transfection from malignant cells and patients with cancer, can induce cell transformation in vitro, as well as tumorigenesis and tumour progression in vivo [14–19]. These studies used cell culture supernatant and plasma/serum for investigating cell transformation and in vivo tumorigenesis. It is known that cfDNA circulates at least in two forms, namely complexed with macromolecules and or with membrane-containing EV. To gain further insight into the role of cfDNA in cell transformation, the present study aimed to demonstrate that cfDNA not associated with EV is responsible for cell transformation and tumorigenesis.

## Materials and methods

### Cell lines and culture

The cell line SW480 (human colon carcinoma, ATCC® CCL-228, RRID: CVCL_0546) and primary murine embryonic fibroblasts NIH3T3 (ATCC® CRL-1658, RRID: CVCL_0594) were obtained from American Type Culture Collection (ATCC®). Cells were cultured in DMEM/F-12 or DMEM (Gibco) supplemented with 10% fetal bovine serum (FBS) (Invitrogen; Thermo Fisher Scientific, Inc.) at 37 °C in a humidified 5% CO_2_ atmosphere.

### Conditioned medium preparation

A total of 2 × 107 SW480 cells were cultured in DMEM/F-12 supplemented with 10% of FBS in 175 cm^2^ flasks (Corning, Inc.). When cells reached 80% confluence, the medium was aspirated, and the flasks were washed with sterile PBS to remove serum components. Next, 20 ml fresh serum-free DMEM/F12 was added and further cultured for 48 h at 37 °C in the presence of 5% CO_2_. After 48 h, the conditioned medium was collected in 50 ml Falcon tubes (Corning Life Sciences) using a sterile pipette.

### Isolation and characterization of structures containing DNA

A total of 40 ml conditioned medium was centrifuged at 1000×*g* for 20 min at 4 °C to pellet the cells in a BIOFUGE PRIMO centrifuge (Thermo Fisher Scientific, Inc.) in 50 ml Falcon tubes (Corning Life Sciences). The resulting conditioned medium, without the pellet, was placed into a new 50 ml Falcon tube and filtered with a 0.45 µm pore size cellulose membrane (25 mm; Millex; MilliporeSigma) to remove cellular debris. The supernatant (40 ml) was transferred to polycarbonate bottles appropriate for the ultracentrifugation rotor. Ultracentrifugation was performed at 120,000×*g* in an ultracentrifuge (Beckman Coulter, Inc.) for 120 min at 4 °C. The resulting supernatant was removed with a pipette without touching the pellet and concentrated to 500 µl with an Amicon stirred cell (8010; Amicon MilliporeSigma) using a cellulose disc of 10 kDa pore size (MilliporeSigma). On the other hand, the pellet obtained was washed twice in PBS, resuspended in 500 µl PBS and stored at − 80 °C until use. The concentrated supernatant, called soluble fraction (SF), was transferred to Eppendorf tubes and stored at − 80 °C until use, while the pellet, called extracellular vesicular fraction (EVF), was resuspended in 500 µl PBS and stored at − 80 °C until use (Fig. Supp. 1A). In summary, for every 40 ml fresh conditioned medium, the final products were 500 µl SF and 500 µl EVF.

### Western blotting

Protein quantification was performed by the Bradford method on both fractions (SF and EVF). In total, 20 µg protein of each fraction (SF and EVF) were separated using 10% SDS-PAGE and transferred onto polyvinylidene fluoride (PVDF) membranes (Bio-Rad Laboratories, Inc.). The membrane was incubated for 1 h with a blocking solution [TBS-Tween-20 (TBS-T), 5% of non-fat milk] and incubated overnight with the specific primary antibody in the blocking solution (dilution 1:1,000). The membranes were washed with TBS-T, and incubated for 1 h with a secondary peroxidase-conjugated antibody anti-mouse IgG-R sc-2300, Santa Cruz Biotechnology, Inc. RRID: AB_641174) at a dilution of 1:1,000, and developed with chemiluminescence substrate (cat. no. WBLUR0100; MilliporeSigma). The primary antibodies used were anti-CD81 (cat. no. sc‐166029; Santa Cruz Biotechnology, Inc. RRID: AB_2275892) and anti-CD9 (cat. no. sc‐13118; Santa Cruz Biotechnology, Inc. RRID: AB_627213).

### Transmission electron microscopy

In total, 100 µl of SF or EVF were mixed with 400 μl fixative containing 2.5% formaldehyde and 2.5% glutaraldehyde (Electron Microscopy Sciences). PBS was exchanged with the fixative solution using an Amicon‐0.5 ml device with a 3 kDa cut‐off filter and concentrated to 100 μl. After 45 min of fixation, a 200‐mesh formvar and carbon-coated grid (Electron Microscopy Sciences) was placed on top of a 7 μl droplet of SF and EVF samples for 20 min. Next, the grid was transferred 7 times onto drops of water for 2 min. Negative staining was performed with 4% uranyl acetate for 12 min. Grids were imaged using a JEOL JEM‐1010 electron microscope equipped with an AMT digital camera (JEOL, Ltd.). Sample preparation and analysis were performed in triplicate.

### DNA isolation and quantitation

cfDNA extraction was performed by SDS/proteinase K digestion followed by phenol/chloroform extraction. DNA extraction started from 500 µl of either SF or EVF, which was mixed with 500 µl SDS/proteinase K solution (Invitrogen; Thermo Fisher Scientific, Inc.), and incubated overnight at 55 °C. An equal volume of phenol/chloroform (1∶1 v/v) was then added, vortexed briefly, and centrifuged at 800×*g* for 10 min (Heraeus Primo R; Thermo Fisher Scientific, Inc.). The aqueous phase was recovered, mixed with an equal volume of chloroform, and centrifuged at 800×*g* (Heraeus Primo R; Thermo Fisher Scientific, Inc.) for 5 min. The aqueous phase was precipitated overnight at − 20 °C with a 1/10 volume of 7.5 M ammonium acetate, 1 µl glycogen, and 2.5 volume of 100% ethanol, and then centrifuged at 1,200 × g (Heraeus Primo R; Thermo Fisher Scientific, Inc.) for 45 min. The DNA pellet was washed with 70% ethanol, air-dried, and resuspended in 20 µl water. Total DNA was quantified with Quant-iT™ PicoGreen® dsDNA Assay Kit (cat. no. P7589; Thermo Fisher Scientific, Inc.) following the manufacturer's instructions. A total of 5 ml PicoGreen® reagent diluted 400-fold was mixed with 1X TE buffer and 5 µl resuspended DNA. After mixing for 3 min, the fluorescence was measured in a Qubit™ 4 Fluorometer (cat. no. Q33226; Invitrogen; Thermo Fisher Scientific, Inc.). Calibration curves were constructed with a range of known concentrations of λ phage DNA solution (Invitrogen; Thermo Fisher Scientific, Inc.).

### Passive transfection and transformation assays

NIH3T3 murine cells, as recipients in the transformation assays, were seeded in 6-well plates (5 × 10^4^ cells per well) and cultured with DMEM supplemented with 10% FBS for 24 h. Subsequently, the medium was removed, and 500 μl fresh DMEM with 2% FBS was added, plus either 500 μl SF, 500 μl EVF or 500 μl reconstituted fraction (a mixture of 250 μl SF/250 μl EVF). As a negative control, cells cultured with 1 ml NHI3T3 conditioned medium (DMEM with 2% FBS) were used, whereas the positive control was performed with 1 ml conditioned medium (DMEM/F-12 with 2% FBS) of SW480 cells (Fig. Supp. 1B). The transformation assay lasted 28 days and the medium was changed every 24 h.

### Morphological analysis

After 28 days of exposure (passive transfection), NIH3T3 cells were examined for foci formation and counted under phase-contrast microscopy. Five foci were cloned from the plates for each condition and expanded under standard conditions for 15 days. Next, the cells were analyzed for the presence of mutated human *KRAS* sequences by PCR. Experiments were performed in triplicates.

### Cell proliferation in soft agar

A pool of cells from *KRAS* sequence-positive foci was used for clonogenic assays in soft agar. After trypsinization, cells were suspended in DMEM containing 0.3% noble agar and 15% FBS. A layer of this suspension was plated on top of a layer of medium containing 0.7% agar without serum. Cells were plated at a density of 1.2 × 10^4^ cells per 2.5 cm^2^ dish. Colonies were scored after 14 days of culture. A colony was defined as containing ≥ 50 cells. Colonies were cloned from agar culture and expanded under standard culture conditions in plastic plates. Next, the cells were analyzed for the presence of mutated human *KRAS* sequences by PCR.

### In vivo experiments

A pool of cells was generated from the soft agar colonies that were positive for the *KRAS* sequence. Tumorigenesis assays were performed in 12-week-old NOD-SCID male mice (Institute of Biomedical Research; UNAM) which were housed in well-ventilated cages under light–dark cycles in a room with standard relative humidity and temperature of 23 °C. Five groups of 8 mice (40 mice in total) were injected subcutaneously in the flank with 2 × 10^6^ cells suspended in 100 µl FBS-free DMEM. We adhered to human endpoints according to the OECD definition (https://www.humane-endpoints.info/en/oecd). Ethics approval was obtained from the Institutional Research Ethics Board and Animal Care Committee of the Instituto Nacional de Cancerologia, Mexico (018/070/IBI) (CEI/1217/17).

Accordingly, visual and clinical inspection was done every other day throughout the experimental time to detect pain, distress, suffering, or impending death. Animals were observed for the following clinical signs: abnormal vocalization, abnormal aggressiveness, abnormal posture, abnormal reaction to handling, abnormal movements, self-induced trauma, open wounds or skin ulceration, difficulties in respiration, corneal ulceration, bone fractures, reluctance to move, abnormal external appearance, rapid weight loss or emaciation or severe dehydration, significant bleeding, or any other factor that suggests that the animal may be in pain or distress. Consequently, the maximum size of tumour volume allowed was ≤ 2500 mm^3^ (to reduce the chance of observing a human endpoint). Tumour size was measured with an electronic calliper, and tumour size-volume was estimated using the formula: V (mm^3^) = (π/6)(1.69)(a × b)^3/2^ (where a is the long diameter; b is the short diameter, and V is the volume)[20]. No anaesthesia method was contemplated in the study. Mice were euthanized in a CO_2_ chamber using 70% vol/min of fill rate (displacement volume). Death was confirmed by the absence of respiration for 3 min followed by cervical dislocation.

### PCR assay

All reactions were performed in 20 µl containing 100 ng template DNA, 10 mmol/l Tris–HCl (pH 8.3), 40 mmol/l KCl, 2 mmol/l MgCl_2_, 200 µmol/l each dNTP, 0.25 U Taq polymerase (Applied Biosystems; Thermo Fisher Scientific, Inc.), and 1 µmol/l each specific primer: Human *KRAS* (5ʹ-GACTGAATATAAACTTGTGGTAGT-3ʹ and 3ʹ-GGACGAATATGATCCAACAATAG-5ʹ), 107-bp amplicon. An initial denaturation at 94 °C for 5 min was followed by 40 cycles of amplification and a final extension step of 5 min at 72 °C. The cycles included denaturation at 94 °C for 30 s and 30 s of annealing at 60 °C. PCRs were carried out in a 2400 thermal cycler (Applied Biosystems; Thermo Fisher Scientific, Inc.). The amplification products were verified by agarose gel electrophoresis.

### Enzymatic digestion followed by passive transfection and transformation assays

After quantifying the concentration of total protein in the SF fraction (500 μl) by Bradford assay, enzymatic digestion assays were carried out. Briefly, protein digestion was performed with 1.5 U proteinase K per mg total protein contained in the SF for 60 min at 37 °C, followed by inactivation at 80 °C for 20 min. For DNase I, the enzyme concentration was 12 U (3.6 Kunitz) per 12 mg DNA content in the SF for 60 min at 37 °C and then inactivation at 65 °C for 15 min. Digestion with both enzymes was performed with the same concentration and times, but digesting first with proteinase K and then with DNase I. After digestion, the passive transfection was carried out as aforementioned.

### Fluorescence staining and cell imaging

For DNA tagging, 500 µl SF was stained with the Quant-iT PicoGreen dye at a ratio of 1:200 using Quant-iT stock (cat. no. P7581; Quant-iT™ PicoGreen™ dsDNA Reagent; Invitrogen; Thermo Fisher Scientific, Inc.), and incubated 30 min at 37 °C. Subsequently, the stained SF was added and incubated at different times (≤ 80 min) to NHI3T3 cells cultured for 24 h over slides in 6-well culture plates (Corning, Inc.). Probes with PBS were used as controls. After exposure, cells were washed three times with 500 µl PBS, mounted with Prolong Diamond Antifade Mounting (Molecular Probes; Thermo Fisher Scientific, Inc.), and analyzed with a confocal microscope (LSM 710 DUO; Zeiss GmbH) with lasers exhibiting excitation wavelengths at 488 and 594 nm. In total, 20 fields were observed for each treatment, and representative images were acquired. The data from three independent experiments were collected with a 63X objective oil immersion lens.

### Cell viability

NIH3T3 cells (5 × 10^4^) were seeded in 6-well plates (Corning, Inc.) and treated with different fractions of SW480 conditioned medium as aforementioned. After 2, 4, 6, and 8 days, the cells were stained with 0.4% trypan blue and quantified in an automated cell counter (Bio-Rad Laboratories, Inc.). All experiments were carried out in triplicate.

### Cell cycle analysis by flow cytometry

NIH3T3 cells were seeded in 6-well plates (Corning, Inc.) at a density of 5 × 10^4^ cells per well. The cells were treated with different fractions of SW480 conditioned medium as aforementioned. After 2, 4, 6, and 8 days, cells were harvested and stained with propidium iodide for 1 h (Sigma-Aldrich; Merck KGaA). In total, 20,000 cells per sample were analyzed using BD FACSCanto™ II flow cytometer (BD Biosciences). Cell cycle analysis was performed with ModFit LT v2.0 software (Verity Software House, Inc.).

### Statistical analysis

Data were represented as the mean ± SEM using GraphPad Prism (version 8.4.2; GraphPad Software; San Diego, CA, USA). Data were explored for normality using Kolmogorov–Smirnov and Shapiro–Wilk tests, data showed parametric (normal) distribution. Differences were analyzed using Student's t-test for unpaired samples. One-way ANOVA with Tukey correction was performed for cell viability, foci formation, clonogenicity, and enzymatic assays. Two-way ANOVA with Tukey correction was performed to determine if the two independent variables, cell cycle phase, and treatment condition, influenced the percentage of cells in each phase, and to evaluate if there was an interaction between these two variables. Values of *P ≤ 0.05, **P ≤ 0.01, and ***P ≤ 0.001 were considered statistically significant.

## Results

### Isolation of SF and EVF

**Fig. 1:**
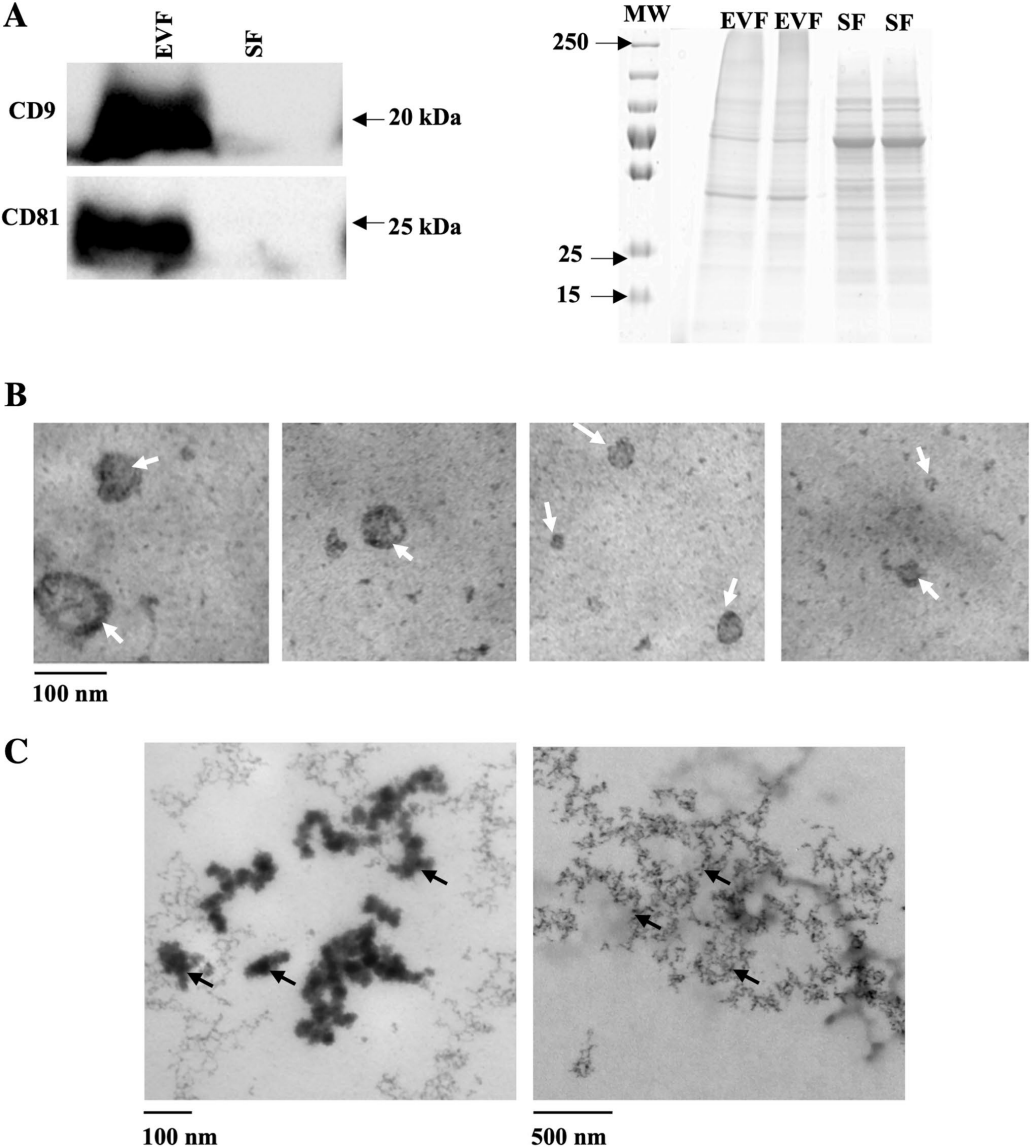
Extracellular DNA structures isolated by ultracentrifugation. **A** Western blotting of protein markers of EV. CD9 and CD81 in EVF and SF samples, and gel staining image of total protein level on blots as a validated loading control (20 μg/well; n = 3). Representative images of **B** EVF and **C** SF derived from SW480 cells and sedimented onto transmission electron microscopy grids for observation. **B** Vesicular round structures with diameters between 40 and 100 nm were observed in the EVF. **C** SF showed long structures of DNA fibers and nucleosome-like structures (black arrow), and other chromatin fibers (black arrow). *SF* soluble fraction, *EVF* fraction associated with EV, *EV* extracellular vesicles

Isolation of SF and EVF. After processing the conditioned medium by ultracentrifugation, the SF and EVF were obtained, and their DNA content was quantified. The DNA concentrations were 18.76 ± 7.7 ng/ml in the EVF and 3.63 ± 1.4 ng/ml in the SF. The presence of the EV membrane markers CD9 and CD81 by western blotting was confirmed in the EVF but not in the SF (Fig. 1A). To further confirm the difference between the two fractions, each sample was processed for electron microscopy. Figure 1B shows vesicular round structures with diameters between 40 and 100 nm in the EVF, but absent in the SF. The SF showed long structures of DNA fibers and nucleosome-like structures (black arrow), and other chromatin fibers of different sizes and fragmented (black arrow) (Fig. 1C).

### Cell transformation and tumorigenesis by cfDNA

**Fig. 2:**
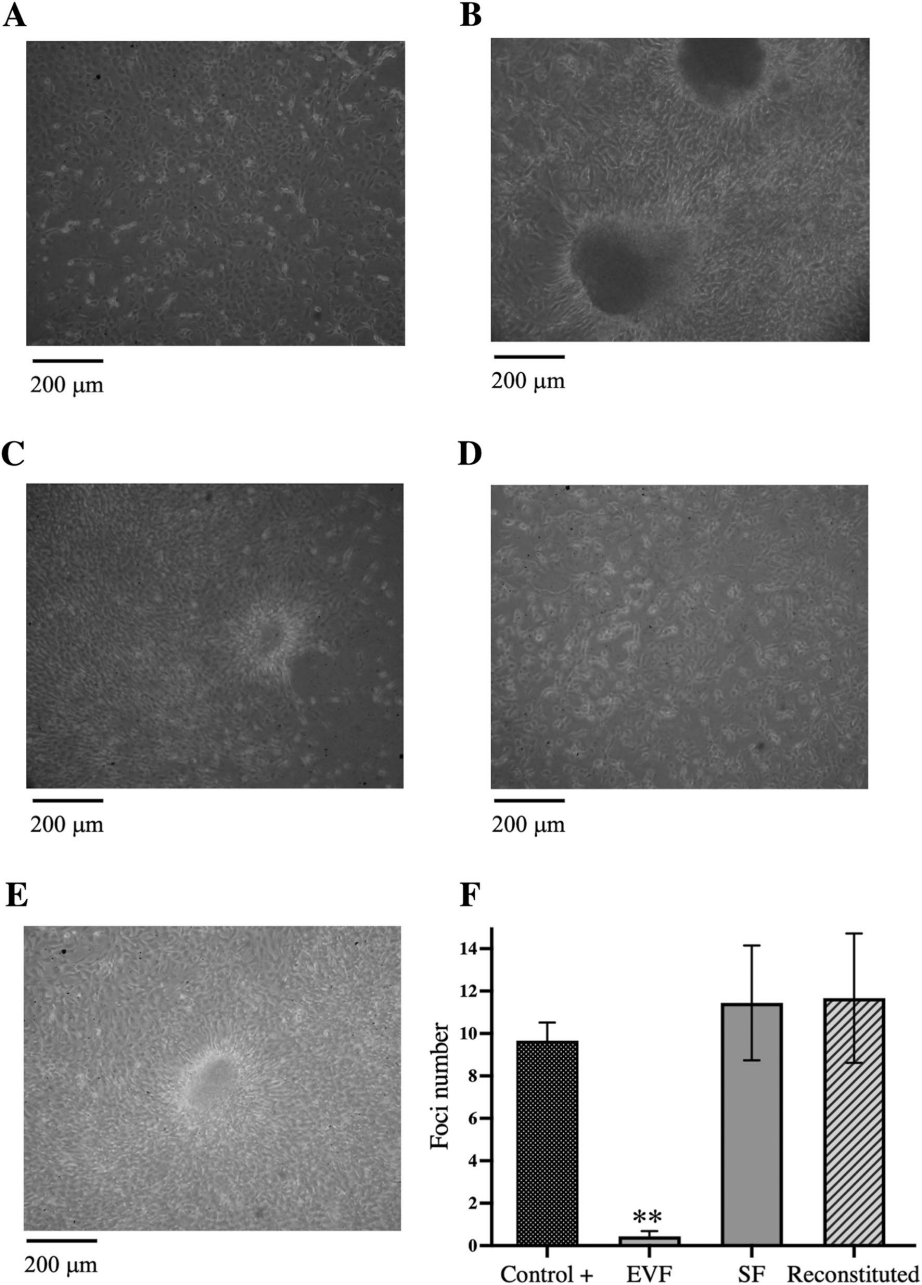
Foci formation in vitro was induced by passive transfection of murine NIH3T3 cells, which were ‘passively’ transformed by exposure to EVF and SF. **A** Culture of NIH3T3 cells under standard conditions. These cells typically displayed contact inhibition and no or rare spontaneous foci formation. **B** Foci of NIH3T3 cells exposed to SW480 conditioned medium as a positive control. **C** Foci formation induced by passive transfection of NIH3T3 cells exposed to SF. **D** Culture of NIH3T3 cells exposed to EVF from SW480 cell conditioned medium. **E** Example of transformed foci of cultured NIH3T3 cells exposed to reconstituted medium from the SW480 cell line. **F** Average number of foci formed by NIH3T3 cells exposed to different conditions. Three independent experiments were performed in triplicate. *SF* soluble fraction, *EVF* fraction associated with EV, *EV* extracellular vesicles

Transformation and tumorigenesis assays include foci formation, proliferation in soft agar, and tumour formation in immunodeficient mice. To evaluate the transforming ability of the cfDNA contained in the EVF and SF, a passive transfection assay was performed where murine NIH3T3 cells were exposed for 28 days to each of the aforementioned fractions. Transformation foci were identified by irregularly shaped, refractile appearance and multilayer growth. Representative photomicrographs are shown in Fig. 2A–E. The mean foci number was 11 ± 3 in the SF, whereas no foci were obtained in cells exposed to the EVF. By contrast, the mean foci number in the positive control (full crude supernatant) was 10 ± 1. Notably, when cells were exposed to the reconstituted fraction of SF plus EVF in equal proportion, the foci numbers were similar to those of SF alone. No foci were observed in NIH3T3 cells (negative control) (Fig. 2A).

**Fig. 3:**
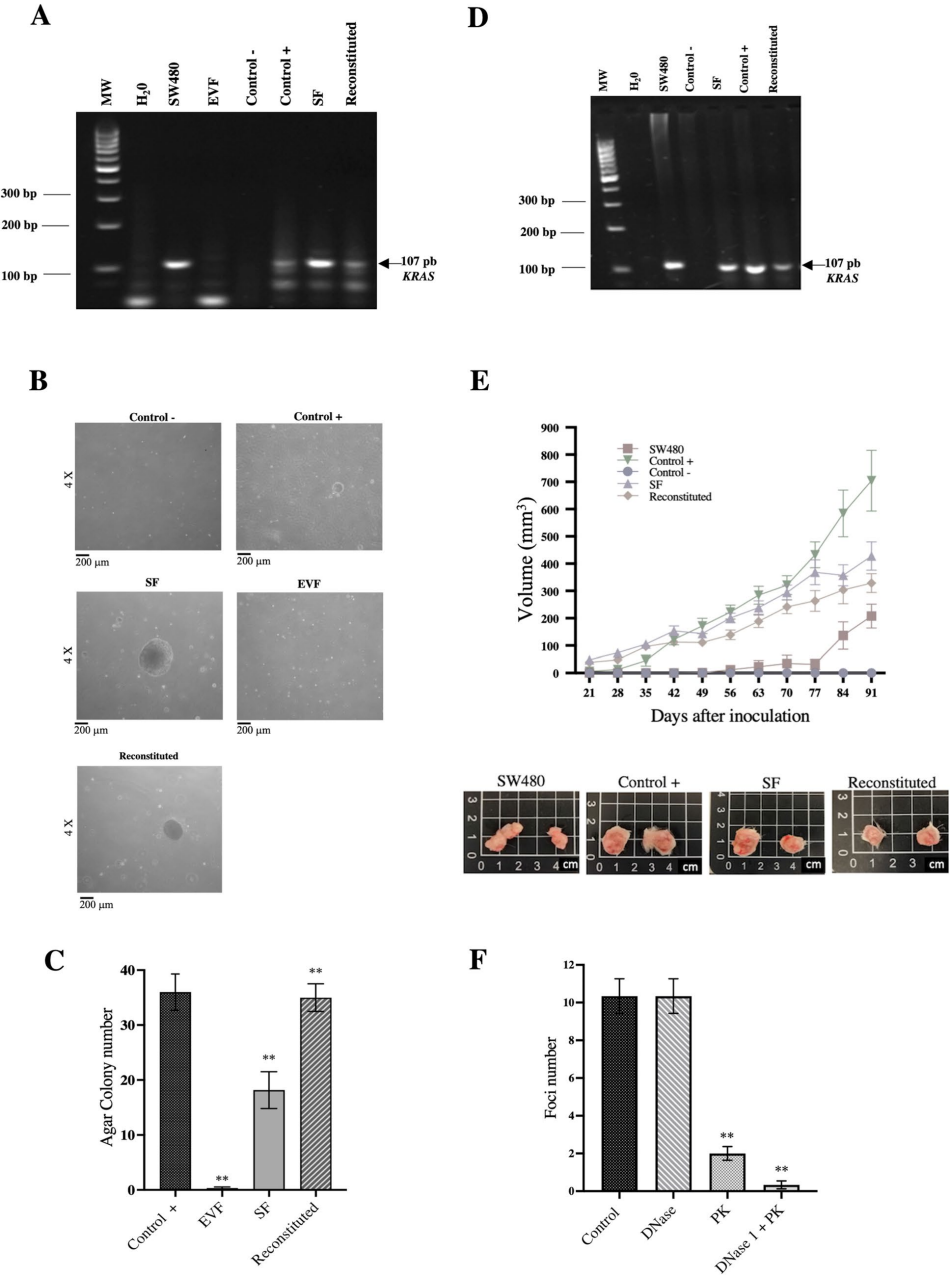
Cell proliferation in agar and tumorigenesis in vivo. **A** PCR products of *KRAS* gene amplification in the genomic DNA of NIH3T3 cells cultured under different supernatant conditions. **B** Colony formation on soft agar by passive transfection of murine NIH3T3 cells with the cfDNA contained in SW480 conditioned medium. **C** Graph representing the average number of colonies formed in NIH3T3 cells cultured under different supernatant conditions; experiments were performed in triplicate. **D** PCR products of *KRAS* gene amplification in genomic DNA of NIH3T3 cells that formed colonies on soft agar after being passively transfected with the cfDNA contained in the fraction associated with extracellular vesicles and SF of SW480 conditioned medium (positive control). **E** Tumor growth curves in SCID mice inoculated with NIH3T3 cells cultured under different supernatant conditions (n = 8) and representative mice tumors. **F** Average number of foci formed by NIH3T3 cells exposed to SF after enzymatic treatment. Three independent experiments were performed in triplicate. cfDNA, extracellular DNA; SF, soluble fraction

The five larger foci of each condition were manually cloned and individually expanded in culture to determine whether cell transformation was associated with DNA transfer. Genomic DNA from cells was extracted and subjected to PCR for *KRAS* analysis. Transformed cells exposed to SF and reconstituted SF + EVF were positive (3 out of 5, respectively), as was the positive control (NIH3T3 cells exposed to SW480 cell supernatant; 4 out of 5), as shown in Fig. 3A. Since no transformed foci were observed in the negative control or EVF condition, these cells were pooled for PCR analyses.

Next, to analyze proliferation in soft agar, the *KRAS*-positive foci from each condition were pooled and further expanded. These cells and the corresponding controls were cultured in soft agar. The results showed 17 ± 4 colonies in the cells transformed with the SF, whereas the number in the positive control was 37 ± 3. The number of colonies in reconstituted (SF/EVS)-treated cells was similar to that of the positive control (37 ± 4) and doubled the number of colonies formed with SF-only transformed cells (Fig. 3B, C).

To assess whether these cells were still harbouring the *KRAS* sequence, five of the largest colonies growing in soft agar were expanded and analyzed again for *KRAS* by PCR. Figure 3D shows that the five colonies from NIH3T3 exposed to SW480 cell supernatant (positive control), the five colonies from the SF, and the five colonies from the reconstituted fraction were positive for *KRAS.* These five colonies from each condition were mixed and expanded in vitro and then injected into mice to investigate the ability to form tumours in immunodeficient mice. The results in Fig. 3E showed tumour growth from cells transformed with SF, reconstituted, and SW480 conditioned medium with no statistically significant differences among the three groups. No growth was observed in parental NIH3T3 cells, whereas faster developing and larger tumours were observed with parental SW480 cells.

### Enzymatic digestion of SF and cell transformation

To determine whether the cfDNA of the SF is protected from enzyme degradation, the SF was digested with DNase I, proteinase K, or both (first proteinase K followed by DNase I). After digestion, the passive transfection was carried out as aforementioned. The results in Fig. 3F showed that treatment with DNase I alone was insufficient to prevent cell transformation (10 ± 2), which was identical to the untreated full supernatant (10 ± 2). On the contrary, treatment with proteinase K only led to a statistically significant decrease in the number of foci (2 ± 1). However, treatment with both enzymes completely abolished the transforming ability of the SF.

### Cell internalization of the cfDNA from the SF

**Fig. 4:**
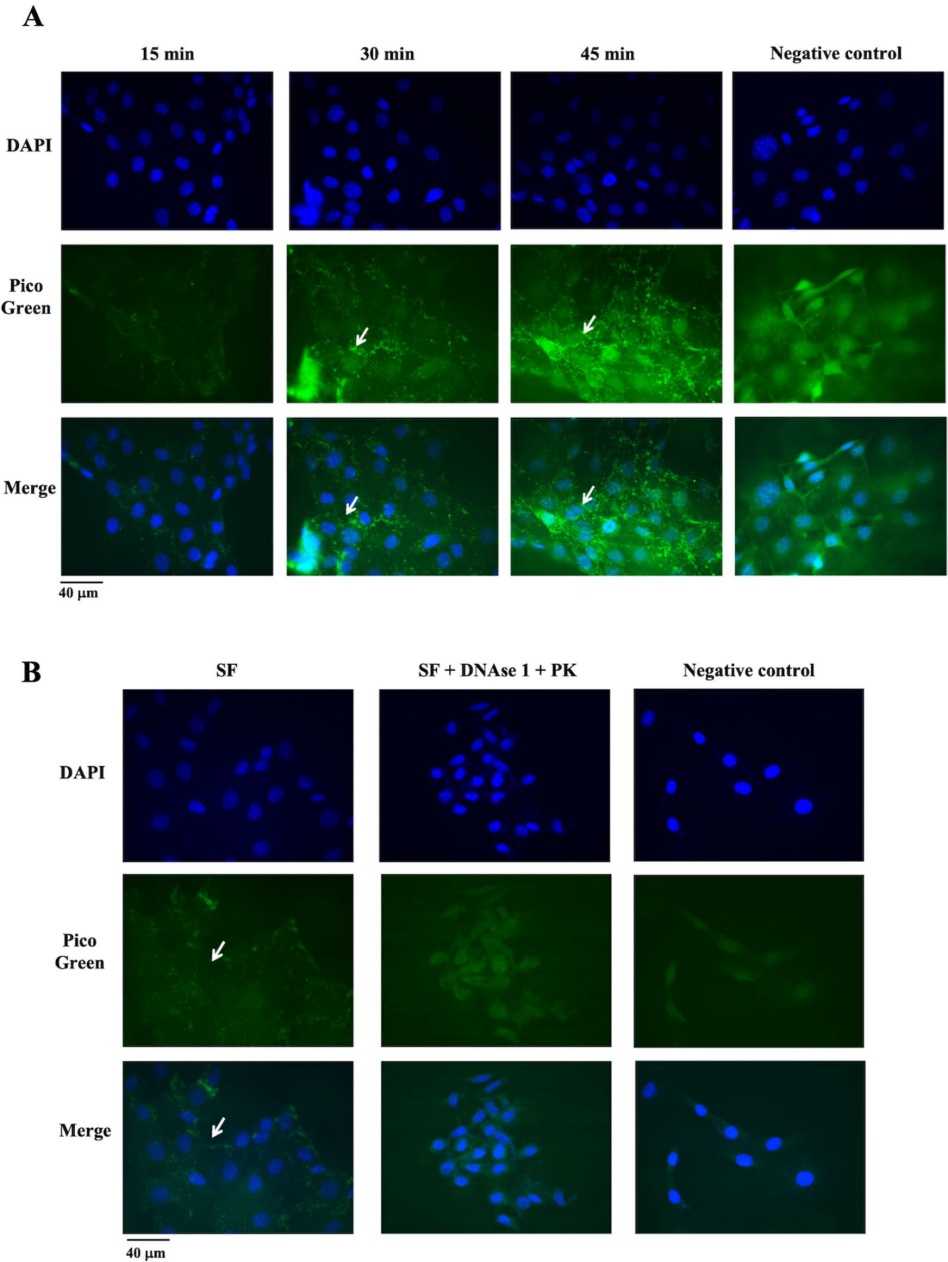
Confocal microscopy images of cfDNA internalization. **A** NIH3T3 cells exposed to cfDNA stained with PicoGreen®. **B** NIH3T3 cells cultured with SF treated with proteinase K and DNase I, stained with Picogreen. Blue, DAPI; green, PicoGreen®. White arrows indicate the aggregates in the cells cytoplasm. cfDNA, extracellular DNA

The mutant KRAS sequence determined by PCR was demonstrated to be transferred to recipient NIH3T3 cells. To visualize the internalization of cfDNA, the SF was stained with the fluorescent probe PicoGreen™, which intercalates into the double-strand DNA and then added to NIH3T3 cells. As shown in Fig. 4A, at 30 min post-incubation, the cells began to incorporate the cfDNA contained in the SF, which was observed as aggregates in the cell cytoplasm. At 45 min post-incubation, the aggregates were more abundant, which was not observed in the control. The presence of fluorescent clusters was corroborated by confocal microscopy. It was observed that these aggregates were at the same focal distance as the nucleus, suggesting incorporation into the cell's cytoplasm. To corroborate that the fluorescent aggregates were DNA, the SF fraction was treated with a mixture of DNase I and proteinase K. After treatment, the fraction was stained with PicoGreen™ and exposed to the NIH3T3 cells for 45 min. As shown in Fig. 4B, there were no fluorescent aggregates after enzymatic treatment compared with the undigested control. Moreover, no clustered PicoGreen signals are observed in control cells.

### Effect of EVF and SF on cell viability and cell cycle

**Fig. 5:**
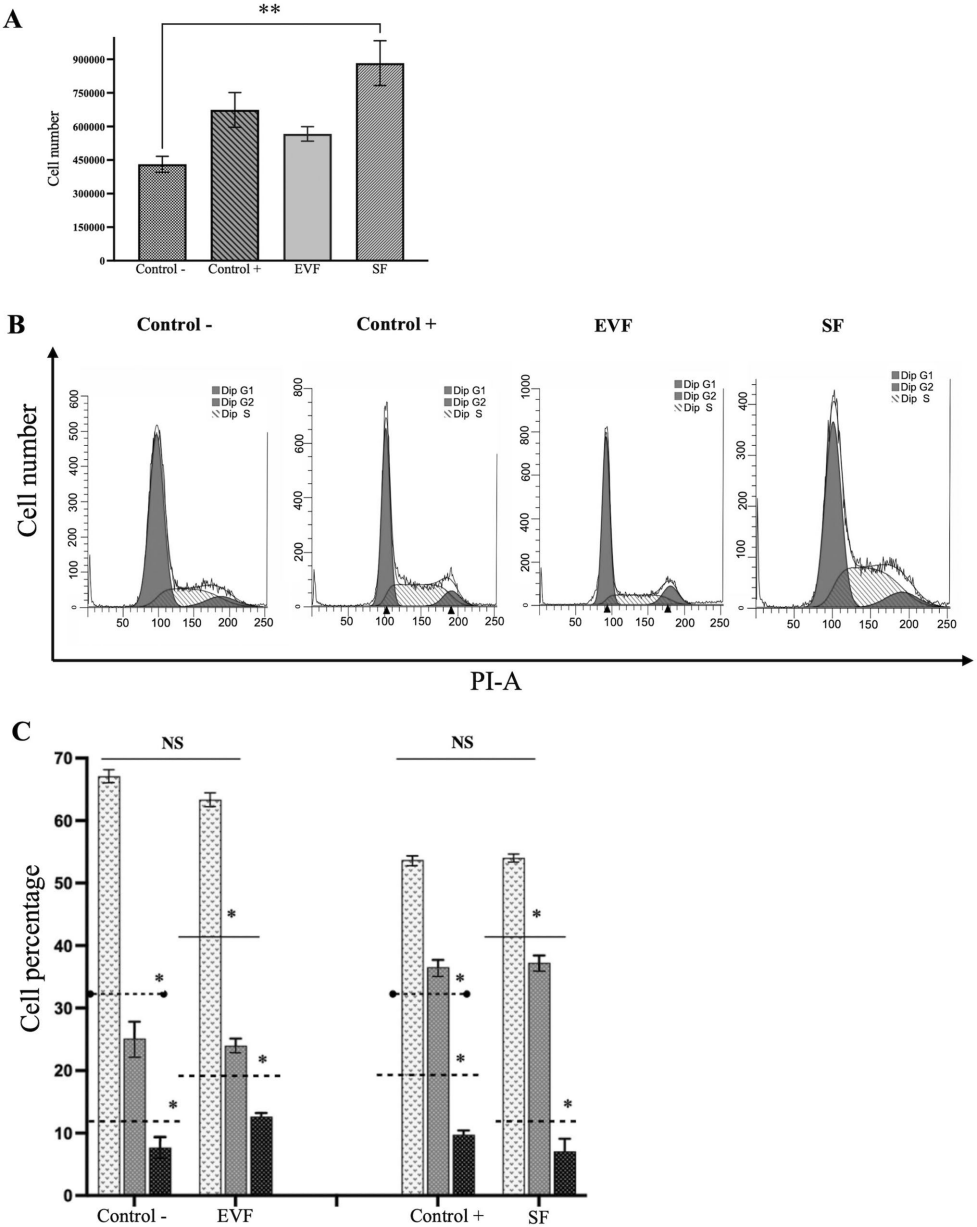
Viability and cell cycle. **A** Viability of NIH3T3 cells treated with different fractions of SW480 conditioned medium. **B** Representative Cell cycle histograms for the four treatment conditions. **C** Viability of NIH3T3 cells treated with different fractions of SW480 conditioned medium. The graphs show the results of 8 days of treatment with the different cell fractions. Asterisks denote statistical significance

To determine whether EVF and SF from the conditioned medium of SW480 cells had any effect on cell viability or cell cycle, NIH3T3 cells were treated with these fractions for 2, 4, 6, and 8 days. Figure 5 shows the results of cell viability and cell cycle of NIH3T3 cells after 8 days of treatment with EVF and SF. As observed in Fig. 5A, cell viability was significantly increased in cells treated with the SF. An increasing trend in viability also occurred in the positive control though, it was not statistically significant. Figure 5B shows the histograms of the cell cycle distribution for the four experimental conditions. There were no significant differences between negative control with EVF and between positive control with SF, indicating that SF is the fraction associated with increased cell proliferation. On the other hand, differences were found between positive control and EVF, negative control with SF, as well between EVF versus SF. P values are shown in Fig. 5C.

## Discussion

The present findings showed that the cfDNA in the supernatant of human malignant colon cancer cells was contained in two fractions, one associated with EV (EVF) and the other not associated with EV (SF). Notably, only the SF could induce cell transformation and tumorigenesis in recipient murine immortalized fibroblasts, which was supported by the cell viability and cell cycle effects of this fraction (Fig). Since cell transformation was prevented by enzymatic digestion with proteinase K and DNAse I, these results confirmed that cell transformation was associated with horizontal DNA transfer between human and murine cells.

cfDNA, more specifically, the one circulating in plasma/serum, may play a role in cancer development and progression beyond its usefulness for liquid biopsy in cancer managemen [21]. Currently, there is experimental demonstration of the in vitro and in vivo transforming abilities of cfDNA. Thus, it is essential to specify which fraction of the cfDNA is responsible for cell transformation.

In 1958, it was shown that nucleic acids extracted from leukemic cells of AKR mice induced multiple tumours in C3H and AKR mice; however, it could not be determined if there were viral particles in these preparations causing tumorigenesis [22–24]. A year later, it was shown that the infectious particle capable of demonstrating cytopathogenicity was the purified DNA and not viral particles [25] Up to the present, it has been concluded undoubtedly that cfDNA induces tumorigenesis associated with horizontal transfer of cfDNA contained in exosomes [26], apoptotic bodies [26], extracellular vesicles [27] the crude supernatant of malignant cell lines [15, 28] and in the plasma/serum of patients with cancer [17, 18, 29, 30].

As horizontal DNA transfer causes cellular transformation and tumorigenesis, it is essential to determine which component of the supernatant or serum/plasma is responsible for the phenomenon and if the transforming cfDNA is or is not contained in EVs. Previous studies have reported that EVs contain cfDNA [6, 7]. EVs comprise a diverse population of biological particles ranging from 30 to 1000 nm [8, 9]. Depending on their origin, biogenesis, and size, EVs are categorized into exosomes, microvesicles, microparticles, and apoptotic bodies [10]. Among these, exosomes typically being defined as small vesicles, 40–150 nm in size, arising from a multivesicular endosomal (MVE) pathway are the most studied, however, their detailed characterization has proven elusive because of their heterogeneity and the use of non-specific isolation techniques. A recent study isolated “pure” classical exosomes bearing the canonical tetraspanin markers CD63, CD81, and CD9 using high-resolution density-gradient fractionation to separate sEVs from non-vesicular material followed by separation of exosomes from other non-exosomal sEVs and by direct immunoaffinity capture (DIC). This two-step procedure was done after removing cell debris and large EVs. Interestingly, the study demonstrated that double-stranded cfDNA is not associated with either classical exosomes or with any EVs [31].

Our data showing that the SF but not the EVF-induced cell transformation and tumorigenesis support these findings, and at the same time contradict previous studies showing that EVs and exosomes were responsible for inducing the malignant phenotype. For instance, EVs containing the bcr/abl sequence induced chronic myeloid leukemia 'like' in animals injected intravenously with these EVs [32]. Likewise, EVs from HT29 colon cancer cell supernatant and serum of patients with colon cancer induced transformation in BRCA1-knockout fibroblasts in vitro [16, 27], and EV from osteosarcoma culture induce cell transformation of embryonic murine fibroblasts [33]. On the other hand, human BRCA1 knockout (BRCA1-KO) fibroblast cells treated with exosomes isolated from the serum of cancer patients underwent cell transformation and formed tumours when transplanted into immunodeficient mice. Interestingly, these tumours growing in immunodeficient mice were carcinomas differentiated into the same lineage of the primary tumours of blood donors [16] Of note, we found higher DNA concentration in the EVF as compared to the SF, most likely however this occurred because of did not pretreat the EVF with DNAse, therefore what we quantified was the DNA contained in the EVF and not the exosome itself as it was shown in the Jeppesen study [31].

he study of horizontal transfer of cfDNA is of enormous relevance due to its therapeutic implications. cfDNA and other macromolecules, specifically RNAs and a wide variety of signalling proteins, and DNA and RNA-associated proteins are not only associated with horizontal transformation and tumorigenesis but with many of the processes of carcinogenesis, including treatment resistance angiogenesis., metastasis, and the antitumor immune response [34–36]. The fact that it is the non-vesicular fraction that contains the cfDNA and that the dsDNA present in NV fractions was highly susceptible to digestion by DNase I [31] supports the observations of the antitumor and antimetastatic effect of systemically administered DNase [37–39]. On the other hand, our study demonstrates that using both proteinase K and DNase I is necessary to prevent transformation and tumorigenesis. In previous works by our group, we have shown that the sole use of DNase I, although it partially degrades the DNA contained in the supernatant or serum, cannot entirely inhibit transformation and tumorigenesis [19, 40]. These data do not contradict the results of Jeppensen, who demonstrated that the cfDNA contained in the non-vesicular fraction is entirely degraded by DNase I since they digested the purified non-vesicular preparation while in our works, the supernatant or serum is used unmodified. This undoubtedly requires further studies. These data do not contradict the results of Jeppensen, who demonstrated that the cfDNA contained in the non-vesicular fraction is entirely degraded by DNase I since they digested the purified non-vesicular preparation while in our works, the supernatant or serum is used unmodified. This undoubtedly requires further studies.

previous studies on the isolation of cfDNA from plasma/serum and supernatants have informed on the isolation of a complex termed virtosome [26]. The virtosome as an elusive entity, contains DNA/RNA-lipoprotein complexes considered a novel cytosolic component of eukaryote cells that, upon its release, can readily enter other cells, where it can modify the biology of the recipient cells, including cell transformation. The isolation process of the SF here reported is similar to that described for obtaining virtosomes from supernatant, plasma or normal rat liver tissue, which is based on 120,000×*g* ultracentrifugation for 120 min [41]. Therefore, the current data support the existence and biological role of the virtosome in cell transformation that can fit the model proposed by Jeppensen et al., of active secretion of cytoplasmic DNA and histones through autophagy- and MVE-dependent, but the exosome-independent mechanism.

A limitation of the present study is the lack of characterization of the macromolecular contained in the SF and the lack of the study of the mechanisms responsible for the transformation. However, the repeated observation that enzymatic treatment with DNAse I and protease prevents transformation has broad implications for the therapeutic potential of cfDNA. Several in vivo studies demonstrated that treatment with DNAse with or without proteases had antitumor effects [37–40, 42, 43]. On the contrary, remarkably, virtosomes obtained from normal or non-replicating cells could negatively affect the proliferation of malignant cell lines [41, 44]. The experimental evidence suggests that a detailed characterization of these cfDNA 'carriers' may shed light on cancer biology and therapeutics.

## Conclusion

In conclusion, the present study showed that the cfDNA contained in the fraction not associated with EV, named SF, has a transforming ability and may correspond to what was previously identified as the virtosome which can be actively secreted by cells through an autophagy- and MVE-dependent, but exosome-independent mechanism. Further studies are required to characterize this complex cfDNA and its mechanism of transformation and tumorigenesis since its manipulation could have therapeutical potential in cancer treatment.

## Supplementary Information

Below is the link to the electronic supplementary material.

Supplementary file1 (DOCX 764 kb)

## Data Availability

The datasets used and/or analyzed during the current study are available from the corresponding author on reasonable request.

## Abbreviations

cfDNA: Cell-free DNA
CirDNA: Circulating DNA
EV: Extracellular vesicles
EVF: Extracellular vesicles fraction
SF: Soluble fraction
