# Supplementary material for: The non-vesicle cell-free DNA (cfDNA) induces cell transformation associated with horizontal DNA transfer
Source: Mol Biol Rep. 2024 Jan 22;51(1):174. doi: 10.1007/s11033-023-09016-w (PMC10803523; doi:10.1007/s11033-023-09016-w)
Supplement: Supplementary file 1 — Supplementary file1 (DOCX 764 kb) [file 11033_2023_9016_MOESM1_ESM.docx]

# Supplementary Material


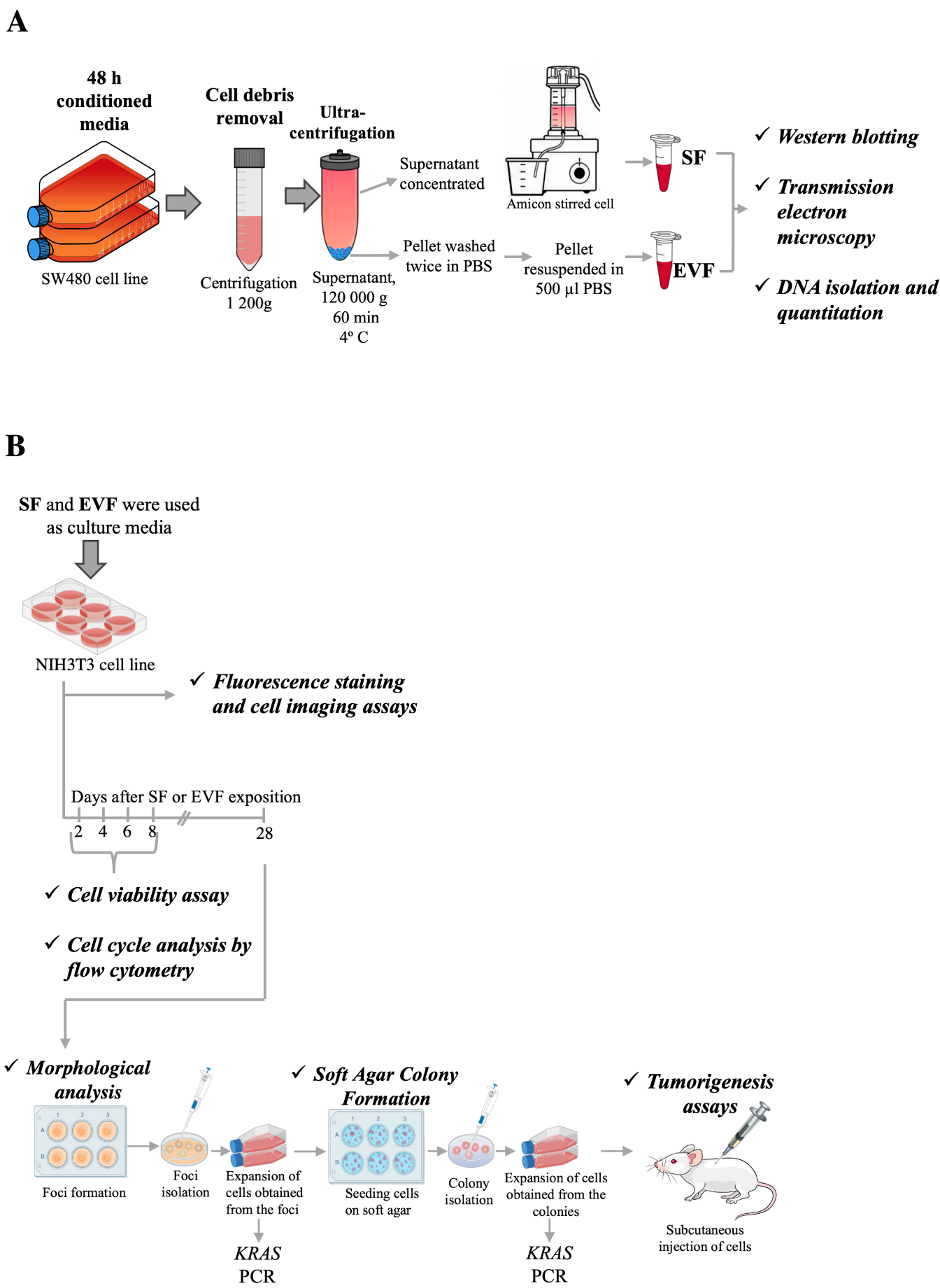
**Fig. S1.** Methodology flowchart. (A) Isolation and characterization of structures containing DNA. (B) Passive transfection and cellular transformation assays.
